# Image-guided protocol with a guide-marker for bone conduction hearing implant placement estimation

**DOI:** 10.1016/j.bjorl.2025.101748

**Published:** 2026-01-07

**Authors:** Renata Tadeu Ramirez Garcia, Antonio Carlos dos Santos, Fabiana Danieli-Hyppolito, Miguel Angelo Hyppolito

**Affiliations:** a Faculdade de Medicina de Ribeirão Preto da Universidade de São Paulo, Department of Ophthalmology, Otorhinolaryngology, Head and Neck Surgery, Ribeirão Preto, SP, Brazil; b Faculdade de Medicina de Ribeirão Preto da Universidade de São Paulo, Center for Imaging Sciences and Medical Physics of the Department of Medical Imaging, Hematology and Clinical Oncology, Ribeirão Preto, SP, Brazil; c Faculdade de Medicina de Ribeirão Preto da Universidade de São Paulo, Department of Health Sciences, RCS, Ribeirão Preto, SP, Brazil

**Keywords:** Bone-anchored hearing aid, Bone thickness, Imaging, Computed tomography, Bone conduction

## Abstract

**Objective:**

To propose a protocol for image-guided implant placement estimation, combining 3D reconstruction of preoperative Computed Tomography (CT) images and a guide-marker, for individuals undergoing percutaneous Bone Conduction Hearing Devices (BCHD).

**Methods:**

Fifty-one subjects aged 6- to 75-years undergoing percutaneous BCHD surgery were included. Three optional implant points were estimated and marked using a guide-marker, and compared with preoperative CT images to define optimal placement. A peer validation was conducted by two independent reviewers to assess the replicability of the protocol in clinical practice.

**Results:**

Bilateral CT images were obtained from all 51 subjects, and a total of 54 BCHD surgeries were performed. Relevant findings, including mastoid cells (19.6%), cranial suture lines (27.5%), perforating vessels (13.7%), bone surface irregularities (1.9%), venous lakes (9.8%), and fixation plates (3.9%) were identified in 52.9% of subjects, and they affected the implant placement in 37.3% of cases. There was moderate agreement between the reviewers regarding implant placement in both right and left ears, and in relation to the actual implant placement performed during the surgical procedure.

**Conclusion:**

The image-guided implant placement estimation, combining 3D reconstruction of preoperative Computed Tomography (CT) images and a guide-marker, has proven reliable and effective for estimating optimal implant placement in subjects undergoing percutaneous BCHD surgery, thereby constituting a valuable tool for clinical practice.

**Level of evidence:**

2.

## Highlights

• Image-guided implant placement estimation was effective to install BCHI.
• The proposed protocol proved to be replicable in clinical practice.
• Relevant findings were identified in 52.9% of subjects, impacting implant placement in 37.3% of cases.
• The proposed protocol may increase the safety and efficacy of BCHD surgery.

## Introduction

Bone Conduction Hearing Devices (BCHDs) are a viable treatment option for intervention of individuals with conductive and mixed hearing loss, or single-sided deafness,^1^ when conventional hearing aids are not indicated or effective. This includes those individuals with cholesteatoma, chronic ear diseases, and various congenital anomalies of the external and middle ear, such as aural atresia and external auditory canal stenosis.^2^

The BCHDs capture sounds and convert them into vibrations, which are transferred through the skull bone directly to the cochlea, regardless of the function of the ear canal and middle ear. In the percutaneous BCHD, the temporal bone stimulation relies on the osseointegration of a 3–4 mm titanium implant. Although it is a surgically straightforward and reliable solution for direct bone stimulation, percutaneous BCHD are associated with challenges related to permanent skin penetration and the need for osteointegration process.^3^

Current recommendations for installing percutaneous Bone Conduction Hearing Implants (BCHI) include placing the implant at a distance of 5–7 cm from the External Auditory Canal (EAC), with the indicator in line with, but not touching, the top of the pinna.^4^ This is intended to ensure that the processor does not touch the ear when attached, and a safe distance from the sigmoid sinus, avoiding risks of bleeding or epidural hematoma.^4^ Still, the selection of the optimal implant placement and length is determined intraoperatively, without the need for preoperative planning.^5^

While this practice is effective to install percutaneous BCHD, it has limitations. The lack of a detailed analysis of cranial vault bone thickness and its anatomical conditions may increase the risk of complications associated with drilling, particularly in regions with high bone thickness variability and/or the presence of cranial sutures and large vessels. Studies^6^^,^^7^ have shown that placing percutaneous BCHI into cranial suture lines, bone marrow, or air cells can compromise osseointegration, leading to implant loss. In addition to osseointegration failures, other complications may occur with percutaneous BCHD, including lateral venous sinus penetration, recurrent infections, soft tissue hypertrophy and/or overgrowth of the implanted abutment, trauma to the implant and surrounding structures.^8^^,^^9^ Intraoperative dura penetration and Cerebrospinal Fluid (CSF) leak, although rare, may also occur during BCHD implantation.^10^

Certain individuals, such as those with craniofacial malformations or previous otologic surgeries, or pediatric patients, are at a heightened risk of experiencing adverse events associated with Bone-anchored Hearing Device (BCHD) implantation.^3^^,^^7^^,^^11^ Notably, individuals with malformations or previous otologic surgeries are more susceptible to dural or lateral sinus exposure during procedure.^7^ Furthermore, studies have reported varying rates of complications related to BCHD in pediatric patients, including failure of osseointegration (0%–14.3%), revision surgery (0%–44.4%), and implant loss (0%–25%).^12^ These findings underscore the importance of careful preoperative planning and consideration of individual patient factors to mitigate the risk of adverse events, especially in these populations.

Furthermore, in the context of percutaneous BCHD, children with syndromic conditions, such as CHARGE or Treacher–Collins, frequently exhibit variable cranial thickness and contour, requiring the implant placement at a more posterior location, typically in the parietal bone, approximately 60–65 mm from the ear canal.^13^ When the required bone thickness is insufficient for installing the BCHI, current practice entails drilling multiple holes, spaced 1 cm apart, until a suitable depth is achieved.^13^ This approach should lead to prolonged surgery time and increased morbidity, particularly in patients with concomitant abnormalities in critical structures.^14^, ^15^, ^16^ Study^17^ showed that 70% of children with Treacher–Collins syndrome required multiple holes for BCHI installation due to irregular skull conformation, demonstrating the need for a more efficient and precise approach in this population. Furthermore, in cases with a history of radical mastoidectomy or cavity obliteration, accurate preoperative planning can be beneficial in determining the feasibility of BCHD placement, or whether alternative options, such as middle ear implants like the Vibrant Soundbridge (VSB), may be more suitable.^18^, ^19^, ^20^

Conventional imaging methods, such as preoperative Computed Tomography (CT) alone, provide limited assistance for BCHD implant placement estimation, as the implant placement relies on the patient's skull contour.^13^ Preoperative surgical planning based on radiologic images, combined with the use of templates, 3D modelling, or markers, has been proposed to estimate the optimal BCHD implant placement. It has shown benefits in ex vivo^21^^,^^22^ and in vivo studies.^3^^,^^13^^,^^23^, ^24^, ^25^, ^26^ However, some of these protocols, such as 3D modelling, are challenging to implement in the clinical routine of BCHD, as the time-consuming nature, availability and cost of 3D modelling are prohibiting factors in its use in many institutions.^13^ Furthermore, most of these protocols are specifically designed for transcutaneous devices, limiting their applicability to percutaneous BCHD.

We propose a preoperative image-guided planning protocol for percutaneous BCHD implant placement estimation, including the use of preoperative CT scans and a guide-marker.^27^ The guide-marker is made of acetate, a flexible and transparent material, and is a simple, fast, and cost-effective tool. It allows visualization of essential anatomical landmarks for precise correspondence between the image-guided estimation and the real BCHI placement, ensuring additional symmetry in bilateral surgeries,^27^ which may be easily replicable and adopted in the surgical routine of percutaneous BCHD implantation.

## Methods

This study was approved by the institutional Research Ethics Committee under registration number 4.387.733.

### Subjects

The inclusion criteria comprised subjects aged over 5-years-old who underwent percutaneous BCHD surgery from January 2020 to December 2021, in a Hospital das Clínicas da Faculdade de Medicina de Ribeirão Preto da USP (FMRP-USP). The exclusion criteria comprised subjects with unilateral hearing loss or those with bilateral hearing loss and asymmetric bone conduction thresholds (i.e., there is more than a 10 dB difference on average across 0.5, 1, 2, and 3 kHz, or 15 dB difference at individual frequencies), with no indication for bilateral surgery. Fifty-one subjects aged from 6- to 75-years-old (Mean = 45.1, SD = ±18.9), both genders (54.9% female, 45.1% male), were included in this study.

### Procedure

**Fig. 1:**
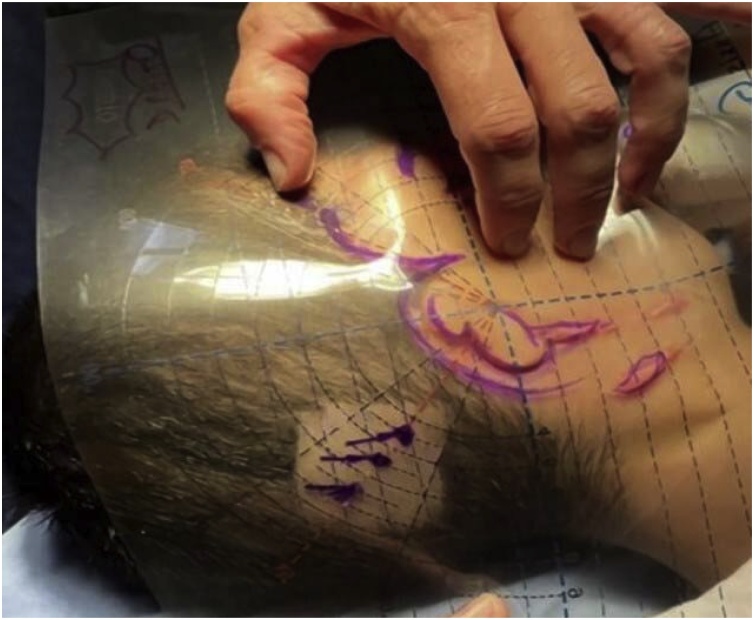
Three optional points (linear distances of 5.5, 6.5 and 7.5 cm to the external auditory canal) estimated via guide-marker for implant placement.

Prior to the surgical procedure, three optional points for BCHI placement were estimated in the subjects, at linear distances of 5.5, 6.5 and 7.5 cm to the EAC, using the guide-marker.^27^ The guide-marker is composed of acetate material, flexible and transparent, which allows the visualization of the necessary anatomical landmarks for choosing the BCHD implant placement (Fig. 1). Although most subjects underwent unilateral BCHD surgery, the procedure was planned and performed bilaterally to ensure optimal and symmetrical implant placement estimation, thereby facilitating potential future bilateral surgery.

**Fig. 2:**
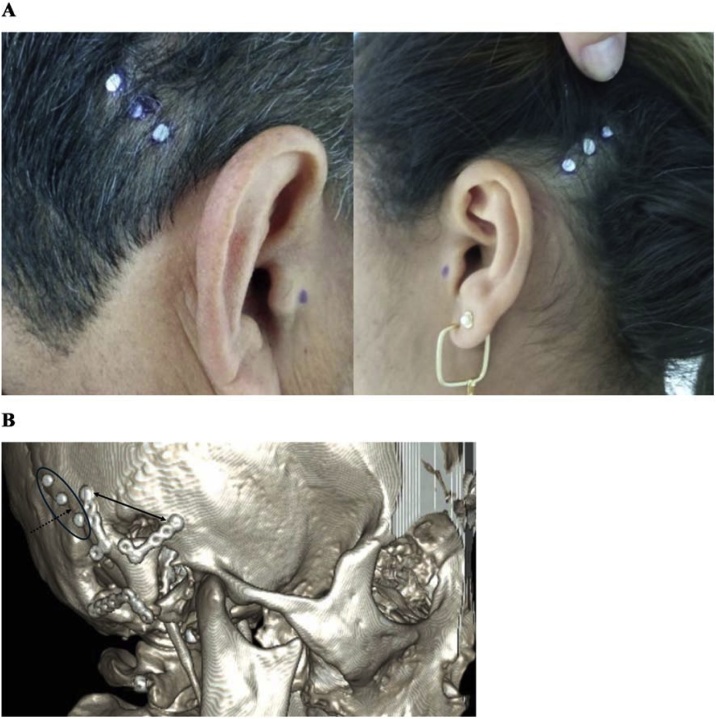
(A) Three optional points for implant placement estimated and marked using Gutta-Percha radiopaque material fixed using a thin layer of elastic collodion. (B) Example of the tridimensional reconstruction of the Computed Tomography (CT) images showing fixation plates (solid arrow) from a previous cranial surgery and the three optional points (circle) marked in a cranial suture line (dotted arrow).

The three estimated optional points were marked using Gutta-Percha radiopaque material fixed to the subjects’ head using a thin layer of elastic collodion (Fig. 2A), and they were considered for choosing the optimal BCHD implant placement, guided by the subsequent preoperative CT images. Fig. 2B shows an example of tridimensional reconstruction of the CT images showing fixation plates from a previous cranial surgery and the three optional points marked in a cranial suture line.

The elastic collodion is composed of pyroxylin, castor oil, alcohol and ether. The alcohol and ether evaporate after application forming a protective film of nitrocellulose and castor pyroxylin, capable of adhering to the surface of the skin up to 7-days. In this study, the CT scans were carried out immediately after (on the same day) the estimation of the optional points for implant placement.

### Image-guided implant placement estimation

The CT temporal bone scans were bilaterally performed in all subjects. CT images were obtained using different systems: the Brillance CT Big Bore 16 (Phillips, Netherlands) and the Aquillion Prime CT 160 (Toshiba, Japan). The Tridimensional (3D) image reconstructions were performed using dedicated workstations: the software Extended Brillance Workspace v.3.5.2.2250 (Philips, Amsterdam, Netherlands) and the Vitrea Enterprise Suite v.6.8.0 (Toshiba, Japan), respectively, both provided by the manufacturers.

The CT imagens were obtained from subjects aiming to provide high-quality information on the anatomy and bone density, thereby mapping the exact location of the surgical site and guiding the estimation of the optimal implant placement. The presence of limited calvarial bone thickness (< 2.5 mm), cranial suture lines, large perforating vessels, venous lakes, mastoid cells and other factors that could compromise the osseointegration were registered and considered negative for the implant placement, thereby avoiding installing BCHI in this optional marked point.

The factors identified through the CT images were compared with direct observations made during the surgical procedure.

### Surgical procedure

All subjects underwent percutaneous BCHD surgery with the Ponto System (Oticon Medical, Askim, Sweden), and minimally invasive punch-only procedures (Minimally Invasive Ponto Surgery/MIPS^28^ and MONO^5^) were performed, since they have routinely been adopted for most BCHD surgery in this hospital, due to their improved postoperative outcomes and invasiveness.^29^^,^^30^ When minimally invasive punch-only procedures were not feasible due to insufficient bone thickness or other limitations, the Linear Incision with Tissue Preservation Technique (LIT-TP)^31^ was employed.

Regardless of the surgical procedure employed, the cannula drill angle was visually adjusted at 90 degrees during bone drilling, in the optimal implant placement marked point, previously determined using the guide-marker and CT-based anatomical landmarks, and considering the contour of the patient's skull.

The cannula plays an important role since it serves as a rigid guide tube, ensuring a perpendicular osteotomy (90 °) to the skull surface, which is critical for proper implant seating and torque distribution. It shields soft tissue from trauma and thermal injury, improving skin preservation and reducing complications. The cannula's snug fit against the periosteum prevents angular deviation during drilling, and its calibrated markings work with depth-stopping drills to control depth and avoid excessive penetration.^5^

### Validation

A peer validation procedure was conducted to assess the replicability of the protocol in clinical practice. Two independent ENT surgeons from the same service, who were not involved in the study and therefore had no prior knowledge of the surgical data (including the implanted ear or implant placement site), were asked to select the optimal marked point for percutaneous BCHD installation based on preoperative CT scans and patient history.

They were provided with a table containing the identification numbers of each subject and their corresponding CT images, which included three optional points for implant placement (Point 1, Point 2, and Point 3, corresponding to linear distances of 5.5, 6.5, and 7.5 mm from the EAC, respectively). The surgeons were then asked to assess the presence or absence of factors that could affect osseointegration and to indicate the optimal point for BCHD implant placement.

Cohen's weighted kappa test^32^ at a significance level of 5% was used to investigate the agreement between the optimal implant placement determined by the ENT surgeons in the peer validation and the real implant placement after the surgical procedure. The following intervals were considered for interpretation^33^: 0 = Poor agreement; 0.01‒0.20 = Slight agreement; 0.21‒0.40 = Fair agreement; 0.41‒0.60 = Moderate agreement; 0.61‒0.80 = Substantial agreement; and 0.81–1 = Almost perfect agreement. The procedures were performed using *R* software version 4.1.1.

## Results

Bilateral CT images were obtained from all 51 subjects for analysis. Two subjects underwent simultaneous bilateral surgery, and one subject had to be reimplanted during the same surgical procedure due to implant instability, reaching 54 BCHD surgeries. Table 1 shows surgical setup and characteristics.

**Table 1:** Surgical setup and characteristics.

Variables	n = 54 (100%)
Implanted ear	Right (n = 25; 46.3%), Left (n = 29; 53.7%)
Surgical technique	LIT-TP (n = 3; 5.5%), MIPS (n = 30; 55.6%), MONO (n = 21; 38.9%)
Implant length	3 mm (n = 4; 7.4%%), 4 mm (n = 50; 92.6%)
Abutment length	6 mm (n = 2; 3.6%), 9 mm (n = 18; 33.4%), 12 mm (n = 34; 63%)
Type of anesthesia	General (n = 3; 5.5%), Local (n = 51; 94.5%)

LI-TP, Linear Incision with Tissue Preservation; MIPS, Minimally Invasive Ponto Surgery; mm, millimeter; n, number of surgeries.

Considering the three optional points estimated for implant placement, based on preoperative CT images, anatomical or other relevant findings were previously identified in 27 of 51 (52.9%) subjects, and they affected the implant placement in 19 (37.3%) surgeries (Table 2). These findings included such as mastoid cells (19.6%), cranial suture lines (27.5%), perforating vessels (13.7%), bone surface irregularities (1.9%), venous lakes (9.8%), and fixation plates (3.9%).

**Table 2:** Surgical and tomographic descriptive data for each subject.

Subjects (n = 51)	Surgeries (n = 54)	Implanted ear (R/L)	Bone thickness ‒ Preop. CT (mm)	Bone tickness ‒ Surgery (mm)	Implant placement (Point 1/2/3)	Anatomical findings	Position of anatomical findings
1	1	R	5	>4	Point 2		
2	2	L	8	>4	Point 2	Mastoid cells	Points 1, 3
3	3	L	9	>4	Point 3	Mastoid cells, cranial suture line	Points 2, 1
4	4	L	7	>4	Point 1	Mastoid cells, perforating vessels	Points 2, 3
5	5	L	8	>4	Point 2	Mastoid cells	Point 1
6	6	L	8	>4	Point 2		
7	7	L	6	>4	Point 1	Cranial suture line	Point 2
8	8	R	7	>4	Point 1	Cranial suture line, perforating vessels	Points 2, 3
9	9	R	8	>4	Point 2		
10	10	L	8	>4	Point 2		
11	11	L	5	>4	Point 1	Perforating vessels, bone surface irregularities	Point 2
12	12	L	7	>4	Point 2		
13	13	R	8	>4	Point 2		
14	14	R	8.4	>4	Point 1		
15	15	R	4.3	>4	Point 2		
15	16	L	5	>4	Point 2		
16	17	L	6	>4	Point 3		
17	18	R	8	>4	Point 2		
18	19	L	7.1	>4	Point 2		
19	20	R	5	>4	Point 1	Cranial suture line	Points 2, 3
20	21	R	7	>4	Point 2		
21	22	L	6	>4	Point 2	Venous lake	Point 1
22	23	L	5	>4	Point 3		
23	24	L	6	>4	Point 3		
24	25	R	6	>4	Point 2		
25	26	L	4	4	Point 3		
26	27	L	8	>4	Point 3		
27	28	R	8	>4	Point 1		
28	29	R	3	3	Point 3	Fixation plates,mastoid cells	Points 1, 2
28	30	R	3	3	Point 3	Fixation plates, mastoid cells	Points 1, 2
29	31	L	8	>4	Point 3		
30	32	L	5	>4	Point 2		
31	33	L	7	>4	Point 3		
32	34	R	9	>4	Point 2	Cranial suture line, mastoid cells	Points 1, 3
33	35	R	6.0	>4	Point 2	Mastoid cells	Point 1
34	36	L	6	>4	Point 1	Cranial suture line	Points 2, 3
35	37	R	4	4	Point 3		
36	38	L	7	>4	Point 2	Cranial suture line	Point 1
37	39	L	4	4	Point 2	Mastoid cells	Point 1
38	40	R	7	>4	Point 2	Venous lake	Point 1
39	41	L	8	>4	Point 1		
40	42	R	7	>4	Point 2		
41	43	L	7	>4	Point 1	Cranial suture line	Points 2, 3
42	44	R	12	>4	Point 2	Perforating vessels	Point 1
43	45	R	6	>4	Point 1		
44	46	L	10	>4	Point 3	Mastoid cells, perforating vessels	Points 1, 2
45	47	R	8	>4	Point 3		
46	48	L	5	>4	Point 3	Cranial suture line, venous lake	Points 1, 2
47	49	R	3	3	Point 2	Cranial suture line, perforating vessels	Points 1, 3
47	50	L	3	3	Point 2	Cranial suture line, perforating vessels	Points 1, 3
48	51	R	5	>4	Point 2	Cranial suture line	Point 1
49	52	L	8	>4	Point 2	Cranial suture line	Point 1
50	53	R	8	>4	Point 1	Venous lake	Point 3
51	54	R	5	>4	Point 2	Cranial suture line, venous lake	Point 1

n, Number of subjects/surgeries; L, Left; R, Right; mm, Millimeters.

The estimated calvarial bone thickness of the subjects, based on preoperative CT images, ranged from 3 to 12 mm (6.52 ± 1.91). There was a correspondence between bone thickness previously estimated and the observations made during the surgical procedure for all subjects. Seven subjects showed calvarial bone thickness ranging between 3 and 4 mm, which could be confirmed intraoperatively with the drill used for the surgeries. For the remaining subjects, the bone thickness was classified as >4 mm, due to the drilling limit imposed by the surgical drill, and they were also confirmed during the surgical procedure (Table 2).

**Fig. 3:**
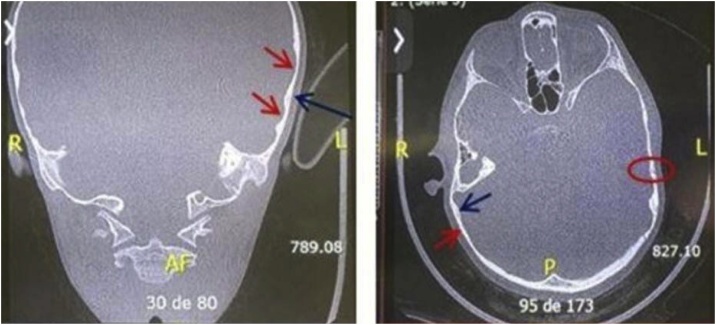
Examples of CT images showing the presence of anatomical factors that could compromise the osseointegration. It is possible to identify bone thickness variability (red arrows), narrow bone thickness (blue arrows) and cranial bone sutures (circle).

**Fig. 4:**
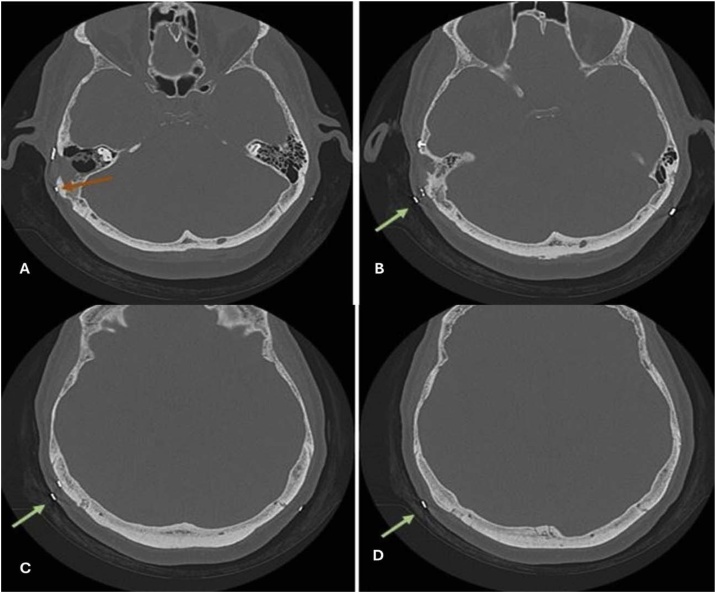
Image-guided implant placement estimation conducted in subject #28. Computed Tomography (CT) showing (A) The fixation screw in the patient's skullcap, at Points 1 and 2; (B) Point 1 showing proximity to veiled mastoid cells; (C) Point 2 showing increased bone thickness; (D) Point 3 showing increased bone thickness. Points 1 and 2 were excluded and Point 3 was selected for implant placement.

Fig. 3 shows examples of CT images with the presence of anatomical factors that could compromise the osseointegration. Fig. 4 shows the image-guided implant placement estimation conducted in subject #28.

The estimation of the optimal implant placement defined by the two reviewers were compared among themselves and with the real implant placement, after surgical procedure (Table 3). There was moderate agreement between the reviewers regarding implant placement in both right and left ears, and in relation to the actual implant placement performed during the surgical procedure.

**Table 3:** Inter-reviewer agreement on optimal implant placement.

			95% CI*		
Reviewers	Comparison	Weighted Kappa	Lower threshold	Upper threshold	p-value
R1 vs. R2	Right ear	0.52	0.27	0.77	0.0001
R1 vs. R2	Left ear	0.60	0.37	0.82	<0.0001
R1	Surgery	0.50	0.23	0.77	0.0003
R2	Surgery	0.58	0.42	0.73	<0.0001

* Cohen’s weighted Kappa concordance test, 95% Confidence Interval.

## Discussion

BCHD were designed to be placed in the calvarial bone, thereby transmitting sound through vibrations from the skull directly to the cochlea. Percutaneous BCHDs rely on a 3–4 mm implant osteointegrated to the skull, and viable bone adjacent to the implant is a prerequisite for a successful outcome.^34^ Therefore, the use of a precise implantation technique is crucial for successful long-term osseointegration.^35^^,^^36^

While bone depth is a crucial factor in determining percutaneous BCHD implant placement, it currently relies on the surgeon's experience. However, this approach may be inaccurate in individuals with complex anatomy, such as those with craniofacial malformations, previous otologic surgeries or even in pediatric patients. Study on 129 percutaneous BCHD installed in pediatric patients showed that 45% of them were in contact with the dura, sigmoid sinus or an air cell, and 6.2% resulted in implant failure.^37^ Other complications, including excessive bleeding, infections, and brain abscesses have also been reported to be related to inadequate bone thickness at the implant site, mainly in pediatric patients.^38^^,^^39^

In this study, we carried out image-guided implant placement estimation in 51 subjects undergoing percutaneous BCHD surgeries, based on the combination of 3D image reconstruction from preoperative CT scans and the use of a guide-marker. Relevant findings were identified in 52.9% of the subjects, and they affected the implant placement in 37.3% of cases. For these subjects, the image-guided protocol enabled a change in the site chosen for BCHD implant placement, thereby increasing surgical precision and safety, and ultimately favoring improved postoperative outcomes.

The image-guided protocol could be easily and rapidly applied to all subjects, allowing for seamless integration into the surgical workflow on the day of surgery. The 3D image reconstruction allowed for accurate estimation of bone thickness at the implant site. There was a high correspondence between the estimated and real bone thickness, confirmed during the surgical procedure. Additionally, anatomical and other relevant findings, including mastoid cells, cranial suture lines, perforating vessels, bone surface irregularities, venous lakes, and fixation plates, at the implant site were precisely identified. The use of the guide-marker enabled precise marking of optimal points for implant placement, based on anatomical landmarks, which were subsequently aligned with the CT image reconstruction. The marked points could be accurately transferred to the contralateral ear, considering bilateral symmetry, which was particularly important for those patients undergoing bilateral BCHD surgery. The guide-marker also enables estimation of implant placement and provision of bilateral symmetry based on alternative anatomical landmarks, particularly for individuals with altered or absent anatomical structures, such as the external auditory canal, due to craniofacial malformations.^27^ Additionally, the guide-marker addressed other individual particularities that may impact device use in daily life, such as glasses or helmet use, which were considered, during implant placement planning, aiming to improve the postoperative results.

There was moderate agreement between the reviewers regarding implant placement in both right and left ears, and in relation to the actual implant placement performed during the surgical procedure. Therefore, the moderate and consistent agreement among the reviewers regarding implant placement reinforces the satisfactory reproducibility of the protocol for the surgical routine of percutaneous BCHD surgeries. However, factors such as differing interpretations of CT findings and choices related to individual subject particularities may have influenced the fact that there was not an even more perfect agreement among them. The selection of the implant placement in subjects undergoing BCHD surgery is complex and involves multiple individual factors. A structured training or calibration session for future reviewers could enhance the level of agreement among them and further standardize the use of this image-guided protocol in future surgeries. Still, the validation procedure should be improved in future studies by incorporating in-person planning that considers patient-specific factors.

A potential limitation of this study is that the sample primarily consists of adults. Given that pediatric patients are more prone to anatomical variations, future studies could benefit from focusing exclusively on pediatric cohorts to better understand the applicability and reproducibility of the protocol in this population.

## Conclusion

The image-guided implant placement estimation, combining 3D reconstruction of preoperative Computed Tomography (CT) images and a guide-marker, has proven reliable and effective for estimating optimal implant placement in subjects undergoing percutaneous BCHD surgery, thereby constituting a valuable tool for clinical practice.

## ORCID ID

Renata Tadeu Ramirez Garcia: 0009-0002-5371-1482

Antonio Carlos Dos Santos: 0000-0002-5502-4734

Fabiana Danieli-Hyppolito: 0000-0002-1078-9602

Miguel Angelo Hyppolito: 0000-0001-9688-782X

## Funding

Coordination for the Improvement of Higher Education Personnel (CAPES) of the Ministry of Education (MEC) of Brazil.

## Declaration of competing interest

RTRG and FD work at Oticon Medical. The remaining authors declare no conflict of interests.
